# A *Trichinella spiralis* Trypsin Drives Macrophage M1 Polarization and Strengthens Cytotoxicity Killing Larvae via Activating the NF‐κB Pathway

**DOI:** 10.1155/tbed/3937439

**Published:** 2026-07-03

**Authors:** Pei Kun Cong, Yao Zhang, Ru Zhang, Xin Zhuo Zhang, Shao Rong Long, Ruo Dan Liu, Jia Xu, Jing Cui, Zhong Quan Wang

**Affiliations:** ^1^ Department of Parasitology, School of Basic Medical Sciences, Zhengzhou University, Zhengzhou, 450001, China, zzu.edu.cn; ^2^ School of Basic Medicine Science, Key Laboratory of Translational Tumor Medicine in Fujian Province, Putian University, Putian City, 351100, China, ptu.edu.cn

**Keywords:** antibody-dependent cellular cytotoxicity (ADCC), macrophage polarization, *Trichinella spiralis*, trypsin

## Abstract

*Trichinella spiralis*, a food‐borne zoonotic parasitic nematode with global distribution, poses a significant hazard to both public health and the safety of animal‐derived meat products. Previous studies have identified a trypsin from *T. spiralis*, designated as TsTryp, within the excretory–secretory antigens (ESA) of intestinal infective larvae (IIL). Notably, the recombinant TsTryp (rTsTryp) was found to facilitate the *T. spiralis* larva invasion of gut epithelia, whereas anti‐rTsTryp antibodies exerted a clear inhibitory effect on this larval invasion process. However, whether TsTryp regulates macrophage polarization in experimental *T. spiralis* infection and its molecular mechanisms are not clear. This present research intends to explore the functional significance of rTsTryp in macrophage polarization and cytotoxicity killing newborn larvae (NBL). The indirect immunofluorescence test verified specific binding between rTsTryp and RAW264.7 cells. qPCR, Western blot, and flow cytometry exhibited that rTsTryp induced the marked upregulation of iNOS expression, increased expression levels of p‐NF‐κB p65 and p‐IκB‐α, and elevated the proportion of CD86^+^ cells and drove the macrophage M1 polarization. qPCR and ELISA results further demonstrated pronounced elevation in proinflammatory cytokine (IL‐6 and TNF‐α) expression levels of macrophages following rTsTryp stimulation. Pretreating macrophages by the NF‐κB inhibitor pyrrolidinecarbodithioic acid (PDTC) significantly abolished and reduced rTsTryp‐increased expression levels of iNOS, p‐NF‐κB p65, and p‐IκB‐α and proinflammatory cytokine production. rTsTryp treatment also prominently strengthened macrophages’ antibody‐dependent cellular cytotoxicity (ADCC) killing NBL, whereas PDTC pretreatment significantly decreased this cytotoxicity. The findings indicated that rTsTryp specifically bound to macrophages and activated the NF‐κB signal pathway, drove M1 polarization and increased proinflammatory cytokine expression, and enhanced the ADCC activity killing larvae.

## 1. Introduction

*Trichinella spiralis* is an important food‐borne parasitic nematode, with human infections from ingestion of raw or inadequately cooked animal meat contaminated by its infective muscle larval (ML) stages [1]. Seventy‐six confirmed trichinellosis cases were officially documented in 11 European Union member nations solely in 2023 [2]. Between 2009 and 2020, eight trichinellosis outbreaks occurred in China, involving 479 cases and 2 deaths, of which seven outbreaks (87.5%) were associated with the consumption of raw or undercooked pork and pork products [3]. Domestic swine‐derived pork serves as the predominant origin of human trichinellosis outbreaks in most of China along with other countries [4, 5]. *Trichinella* infection poses a significant threat to societal public health infrastructure and national food security frameworks. However, due to its multiple natural hosts and the absence of commercially available veterinary anti‐*Trichinella* vaccines, control and elimination of the parasite from food animals remain challenging [6]. Therefore, identifying highly immune‐protective molecules in *Trichinella* proteins and developing an effective preventive vaccine is critical to block *Trichinella* infection in food animals [7, 8].

The invasion of host enteral mucosa by intestinal infective larvae (IIL) constitutes a critical step during the early phase of *T. spiralis* infection. Serine proteases are one vital superfamily of proteolytic enzymes and play a crucial role in protein digestion and larval invasion. Serine proteases are involved in various processes of parasitic nematode infection, including tissue penetration, molting, digestion, and fibrinolysis [9, 10]. Due to the lack of specialized oral structures such as teeth or stylets, *Trichinella* larvae invade the intestinal mucosa not merely through mechanical damage but likely through secreted proteases.

Our earlier research successfully characterized a trypsin homolog from *T. spiralis* (TsTryp, GenBank Accession: XM_003381619.1) within IIL excretory–secretory antigens (ESA) by immunoproteomic approaches, with this TsTryp protein exhibiting abundant expression specifically at the IIL developmental phase [11, 12]. TsTryp is predominantly located within the cuticular layer and stichosomal structure of this parasitic nematode, functioning as both a surface‐associated and a secreted proteolytic enzyme. Recombinant TsTryp (rTsTryp) retains catalytic activity comparable to native trypsin, although it lacks direct hydrolytic capacity against intestinal epithelial tight‐junction (TJ) structural proteins. rTsTryp binds and interacts specifically with protease‐activated receptor‐2 (PAR2) in intestinal mucosa, activated the ERK1/2 pathway, downregulated TJ protein levels, and compromised the enteral epithelial integrity to facilitate larval penetration across enteral mucosal barrier. rTsTryp distinctly promoted larval invasion of gut epithelium; PAR2 antagonist AZ3451, ERK1/2 inhibitor PD98059, and anti‐rTsTryp antibodies obviously impeded the larval invasion [13, 14]. However, whether TsTryp regulates macrophage polarization during the process of *T. spiralis* infection and the underlying mechanism are not clear.

This present work sets out to characterize the functional contribution of rTsTryp in macrophage polarization and cytotoxicity killing newborn larvae (NBL) via antibody‐dependent cellular cytotoxicity (ADCC) mechanisms. The expected results will provide new insights for understanding immune activation profiles triggered at the early stage of *T. spiralis* infection [15–17].

## 2. Materials and Methods

### 2.1. *Trichinella* Isolate, Animal, and Cultured Cell Line

The *T. spiralis* isolate ISS534 employed in this research originated from a naturally infected domestic swine in Henan Province of China and has been serially passaged in BALB/c mice [18]. Female BALB/c mice aged 4–6 weeks were obtained from the Experimental Animal Center of Henan Province (Zhengzhou). RAW264.7 macrophage cells were bought from the Cell Bank of the Chinese Academy of Sciences (Shanghai) and cultured under 37°C with 5% CO_2_ in DMEM medium replenished with 10% fetal bovine serum (FBS), 100 U/mL penicillin, and 100 μg/mL streptomycin [17].

### 2.2. Isolation of *T. spiralis* Worms and Preparation of IIL ESA

The ML was obtained via artificial digestion of the experimentally *T. spiralis*‐infected murine skeletal muscle tissues at 35 days postinfection (dpi) [19]. The ML worms were activated to develop into the IIL stage by incubation with 5% porcine bile (collected from a local abattoir) at 37°C for 2 h [20]. Following being rinsed with sterile physiological saline and serum‐free RPMI 1640 medium supplemented with 100 U/mL penicillin and 100 μg/mL streptomycin, these larvae were cultured at 37°C in a 5% CO_2_ incubator for 18 h. An Amicon Ultra‐3 centrifugal filtration device (MW cut‐off value: 3 kDa) was used to filtrate the supernatant, which was centrifuged at 4°C and 5000 ×*g* for 3 h. The obtained IIL ESA was preserved under −80°C cryogenic conditions for subsequent experimental application [21]. We also harvested adult worms from the gut of infected mice at 6 days postinfection and isolated the NBL via incubating the collected adult worms in a serum‐free 1640 culture medium within a 37°C incubator supplemented with 5% CO_2_ for a continuous period of 72 h [22].

### 2.3. Expression and Purification of rTsTryp

TsTryp has two trypsin‐like functional domains (N‐terminal and C‐terminal). In the present work, the 26 kDa C‐terminal domain of TsTryp was cloned and heterologously expressed. The full‐length TsTryp cDNA sequence was successfully amplified via PCR and inserted into the vector pQE‐80L, which bears an N‐terminal His‐tag (Novagen, USA). The recombinant pQE‐80L/TsTryp was transformed into *Escherichia coli* BL21 (DE3) (Novagen, USA). rTsTryp was expressed with the induction of 0.5 mM isopropylthio‐β‐D‐galactoside (IPTG) at 37°C for 6 h. Purification of the rTsTryp protein was performed via a Ni‐NTA affinity chromatograph with a His‐tag purification kit (Sangon Biotech, Shanghai, China). The purified rTsTryp was characterized via SDS‐PAGE and Western blotting [13].

### 2.4. CCK‐8 Test of Macrophage Cellular Viability

The impacts of rTsTryp and IIL ESA on RAW264.7 macrophage viability were evaluated using the Cell Counting Kit‐8 (CCK‐8) (Epizyme Biotech, Shanghai, China) [23]. To assess the impact of rTsTryp on cell viability, cells were stimulated with varying concentrations (5, 10, 15, 20 and 25 μg/mL) of rTsTryp or IIL ESA for 24 h and 48 h, respectively. Following co‐incubation, the CCK‐8 reagent was supplemented to individual wells, followed by an additional incubation of 2 h. Absorbance values at 450 nm were detected with a multimode microplate reader (SpectraMax i3X; Molecular Devices, USA). Cellular viability was calculated in accordance with the computational formula [16, 17]:

Cell survival rate %=ODexperiment− ODblank/ODcontrol− ODblank×100%.

.

### 2.5. Indirect Immunofluorescence Assay (IIFA) of Binding of rTsTryp to RAW264.7 Cells

IIFA was conducted to ascertain the binding and interaction between rTsTryp and RAW264.7 macrophages, as described in previous studies [24, 25]. Briefly, the cells were seeded on coverslips in a 6‐well plate and cultivated till reaching >80% confluence. After three sequential PBS washes, cells were fixed in 4% paraformaldehyde for 20 min at ambient temperature, followed by incubation with 25 μg/mL rTsTryp at 37°C for 2 h. Cells were then sealed using 5% goat serum at 37°C for 1 h and further incubated with 1:10‐diluted different sera (anti‐rTsTryp immune serum, infection serum, and pre‐immune normal serum) at 37°C for 1 h. Alexa Fluor‐CY3‐conjugated anti‐mouse IgG (1:100; Abways, Shanghai, China) was applied as a secondary antibody, while 4^′^, 6‐diamidino‐2‐phenylindole (DAPI) was utilized for nuclear counterstaining. Finally, a professional fluorescence microscopy system (Olympus, Tokyo, Japan) was used to visualize the fluorescence signal [26].

### 2.6. qPCR and Western Blotting of rTsTryp Driving RAW264.7 Cell M1 Polarization via NF‐κB Pathway

To exclude endotoxin interference in rTsTryp, we used an endotoxin removal kit (Thermo Fisher Scientific, Waltham, USA) in this study. Endotoxin levels in rTsTryp were determined by the limulus amebocyte lysate (LAL) Kit (Bioendo Technologies, Xiamen, China), with results indicating that endotoxin concentration was <0.05 EU/mL, implying that the endotoxin level in rTsTryp had a minimal influence on the macrophages [25]. RAW264.7 cells were stimulated by 25 μg/mL of rTsTryp for 24 h. Lipopolysaccharide (LPS) at 200 ng/mL and interleukin‐4 (IL‐4) at 20 ng/mL acted as the positive control for M1 and M2 polarization, respectively [25, 27]. Moreover, an unrelated protein control (tag protein TRX), a heat‐inactivated denatured rTsTryp, and an enzymatically inactive TsTryp control (e.g., rTsTryp + serine protease‐specific inhibitor PMSF) were also used in this study. Following 24 h of incubation at 37°C and washing, total RNAs and soluble proteins were extracted from these treated cells for qPCR and Western blotting, separately [24, 28].

qPCR was carried out as described before [21, 29]. Briefly, the cells were stimulated with 25 μg/mL rTsTryp for 24 h. Total RNA was isolated from stimulated cells, followed by reverse transcription to synthesize cDNA, and qPCR was carried out with the synthesized cDNA as a template to evaluate the mRNA expression level of M1‐associated markers (iNOS, IL‐6, TNF‐α) and M2‐associated markers (Arg‐1, IL‐10, TGF‐β). DMEM served as the blank control, the ESA as the worm protein, and β‐actin as the internal reference gene [16], and the relative mRNA transcription level of target genes was quantified via the 2^−ΔΔCt^ comparative threshold cycle method [30]. Specific primer sequences for qPCR amplification of murine macrophage markers and cytokines are presented in Table 1.

**Table 1:** Specific primer sequences of mouse macrophage markers and cytokines for qPCR.

Genes	Sequences (5′end to 3′ end)	GenBank number
TNF‐α	F: CCCTCACACTCAGATCATCTTCT	NM_013693.3
	R: GCTACGACGTGGGCTACAG	
TGF‐β	F: AGCAACAATTCCTGGCGTTACCT	NM_011577.2
	R: CCTGTATTCCGTCTCCTTGGTTCA	
IL‐6	F: TACCACTTCACAAGTCGGAGGC	NM_001314054.1
	R: CTGCAAGTGCATCATCGTTGTTC	
IL‐10	F: CCCTTTGCTATGGTGTCCTT	NM_010548.2
	R: TGGTTTCTCTTCCCAAGACC	
iNOS	F: GAGAGACAGGGAAGTCTGAAGCAC	NM_010927.4
	R: CCAGCAGTAGTTGCTCCTCTTC	
Arg‐1	F: CATTGGCTTGCGAGACGTAGAC	NM_007482.3
	R: GCTGAAGGTCTCTTCCATCACC	
β‐actin	F: CTACCTCATGAAGATCCTGACC	NM_007393.5
	R: CACAGCTTCTCTTTGATGTCAC	

Western blotting was conducted based on prior protocols [31]. Soluble proteins were extracted from rTsTryp‐treated RAW264.7 cells, separated by SDS‐PAGE, and subsequently subjected to Western blot analysis [26, 32]. Primary antibodies included antibodies against iNOS (1:5000; Abcam; UK), Arg‐1 (1:5000, Servicebio, China), p‐IκB‐α (1:1000, Abmart, China), p‐NF‐κB p65 (1:1000, Abmart, China), IκB‐α (1:1000, Abmart, China), NF‐κB p65 (1:1000, Abmart, China), and anti‐mouse β‐actin (1:1000, Servicebio, China). Horseradish peroxidase (HRP)‐conjugated secondary antibodies consisted of anti‐rabbit and anti‐mouse antibody IgG. After the strip was rinsed, the protein band was colored with Omni‐ECL reagent (Epizyme, Shanghai, China), and the band intensity was quantified with ImageJ software (National Institutes of Health, USA) to determine the relative expression abundances of the target protein [33].

### 2.7. Flow Cytometry

Flow cytometry of macrophage polarization was carried out as previously reported [17, 25]. In short, RAW264.7 macrophages were plated in a 6‐well plate at a density of 5 × 10^6^ cells per well and grew to adherence for 1 h. After removing nonadhered cells by washes, the adhered cells were treated by rTsTryp for 24 h; LPS or IL‐4 was used as a positive control for M1 or M2 polarization, respectively, while the IIL ESA served as the natural *T. spiralis* antigen control. Following washing, cells were incubated with 1 mL of FACS buffer (100 mL PBS + 0.1% BSA + 0.5 mM EDTA) for 10 min and then gently detached, collected, and counted. The total of 3 × 10^6^ cells was transferred to a 1.5 mL tube, centrifuged at 286 ×*g* for 5 min at 4°C, and the supernatant was removed. The cells were identified with FITC‐anti‐F4/80 antibody conjugate (Biolegend, USA) and PerCP‐cyanine 5.5‐anti‐CD11b antibody conjugate (BioLegend, USA). PE‐anti‐CD86/APC‐anti‐CD206 antibody conjugate (BioLegend, USA) was used separately as the M1/M2 marker. For cellular staining, these cells were fixed and permeabilized using a permeabilization buffer and then incubated for 20 min with APC‐anti‐CD206 conjugate (BioLegend, 1:200) at 4°C. Following the final wash with FACS buffer and centrifugation at 500 ×*g* and 4°C for 10 min, these cells were resuspended in 100 μL FACS buffer. Samples were analyzed with a BD FACS Canto flow cytometer (BD Biosciences, USA), and data were analyzed via FlowJo software (Ashland, USA) [16, 34, 35].

### 2.8. qPCR and ELISA Determination of Cytokine Expression in RAW264.7 Cells Stimulated With rTsTryp

To determine the mRNA level of macrophage cytokines (IL‐6, TNF‐α, IL‐10, and TGF‐β), total RNA was purified from rTsTryp‐treated macrophages, and qPCR was performed [16]. Cell culture supernatants were also collected to assay the contents of the above‐mentioned cytokines by a sandwich ELISA [8]. Briefly, the plate was precoated with purified anti‐mouse antibody (1 μg/mL) overnight at 4°C. The plate was rinsed with PBS‐0.5% Tween‐20 (PBST) and blocked at 37°C for 2 h with 3% FBS in PBST. Following washes again, 50 μL of each sample and serially diluted standards (0.0625, 0.125, 0.25, 0.5, 1, 2, and 4 ng/mL) were dispensed into wells and incubated for 2 h. Following another washing, the plate was incubated with 1 μg/mL biotinylated anti‐mouse antibody in PBST at 37°C for 2 h, then with HRP‐avidin conjugate (diluted to a ratio of 1:5000 in PBST buffer) in a dark place for 1 h. Then, the plates were developed with o‐phenylenediamine dihydrochloride (OPD; Sigma) + 0.15% H_2_O_2_ as the substrate. The reaction was terminated with 2 M H_2_SO_4_, and optical density readings of 492 nm were measured within 10 min using a microplate spectrophotometer. Each sample was analyzed in technical duplicate. A standard curve was generated based on the OD values of serial standards, and the cytokine concentration in each sample was shown as picograms per milliliter (pg/mL) [25, 36].

### 2.9. Assay of Nitric Oxide (NO) Content in Cell Culture Supernatant by Griess Assay

Based on the Griess reaction, accumulated nitrite in the culture medium was detected as an indicator of the NO content [37]. In short, macrophages were treated by rTsTryp or IIL ESA at 37°C for 24 h. LPS/IL‐4 was used as the control of M1/M2 polarization, respectively. Moreover, cells were first pretreated with NF‐κB‐specific inhibitor pyrrolidinecarbodithioic acid (PDTC) for 2 h and then cultured with rTsTryp for 24 h [38]. The culture supernatant was obtained and mixed sequentially with Griess Reagent I and II (Beyotime, Shanghai, China). Room‐temperature incubation was conducted for 5 min, and spectrophotometric quantification of the sample absorbance at 540 nm was performed by a microplate reader. The standard curve was generated with a serial concentration of NaNO_2_ [16].

### 2.10. Antibody‐Dependent Cell‐Mediated Cytotoxicity (ADCC) Assay

To assess the rTsTryp promotion effect on ADCC, RAW264.7 macrophages were plated onto 24‐well plates and then treated with rTsTryp for 24 h. Following incubation, 100 NBL and *T. spiralis*‐infected serum, rTsTryp‐immunized serum, or normal serum (1:100 dilutions) were added to each well, with subsequent continuous incubation lasting 36 h. Larval viability was observed on microscopy and evaluated in light of their morphology and activity. Viable NBL displayed vigorous motility, while the dead NBL was inactive and straight [16, 25]. Cytotoxicity values were determined by computing the proportion of nonviable NBL or macrophage‐attached NBL relative to total larvae in each experimental condition [22, 39].

Additionally, to determine the PDTC inhibitory effect on the macrophage’s cytotoxicity, RAW264.7 cells were first exposed to PDTC for 2 h and then treated with rTsTryp for 24 h, and the ADCC assay was carried out [31, 40].

### 2.11. IIFA of NF‐κB p65 Protein Nuclear Translocation

To verify the function of the NF‐κB signaling pathway in macrophage M1 polarization, IIFA was also performed to assess the NF‐κB p65 protein nuclear translocation in macrophages treated by rTsTryp [25]. Shortly, RAW264.7 macrophages were cultured with rTsTryp for 24 h, with PBS serving as the negative control and LPS acting as the positive control. After fixation and blockage, the macrophages were incubated with anti‐NF‐κB p65 antibody overnight at 4°C. CY3‐goat anti‐mouse IgG conjugate (1:100; Abways) served as a secondary antibody, and DAPI was applied to counterstain cell nuclei. Fluorescence staining was examined on fluorescence microscopy (Olympus).

### 2.12. Statistical Analysis

All statistical evaluations were carried out via SPSS 22.0. Sample data were presented as the mean ± standard deviation (SD). Shapiro–Wilk and Levene’s tests were separately used to evaluate the datum normality and variance homogeneity within the measured samples. One‐way ANOVA, Chi‐square test, or Student’s *t*‐test was served to ascertain statistical differences. *p*  < 0.05 was regarded as statistical significance.

## 3. Results

### 3.1. rTsTryp’s Effect on Cellular Viability of RAW264.7 Macrophages

The impact of rTsTryp and IIL ESA on RAW264.7 cell viability was assessed following 24 and 48 h of incubation. The results revealed that after stimulation for 24 h, both the rTsTryp and IIL ESA groups at concentrations of 5–25 μg/mL did not exhibit any cell cytotoxicity (*F*_rTsTryp_ = 0.8573, *p* = 0.5213; *F*_IIL ESA_ = 6.889, *p* = 0.263). In contrast, treatment with 10–25 μg/mL of rTsTry and IIL ESA for 48 h significantly reduced cell viability (*F*_rTsTryp_ = 20.75, *p* < 0.001; *F*_IIL ESA_ = 4.808, *p* < 0.05) (Supporting Information 1: Figure S1). Accordingly, RAW264.7 cells underwent a 24‐h treatment with 25 μg/mL rTsTryp and IIL ESA in subsequent tests.

### 3.2. rTsTryp Binding to RAW264.7 Cell Membranes

**Figure 1:**
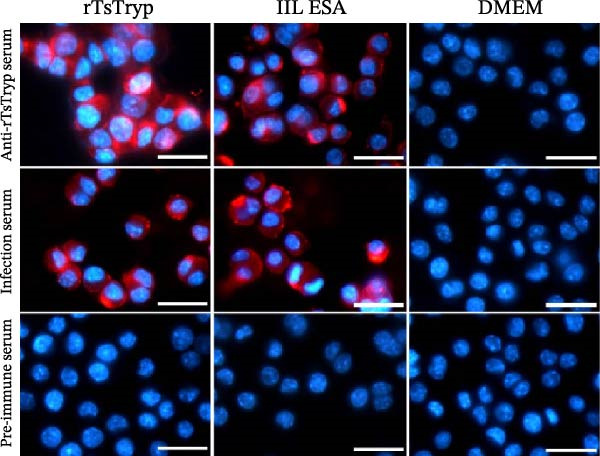
IIFA of rTsTryp binding to RAW264.7 macrophages. RAW264.7 cells were incubated with rTsTryp at 37°C for 2 h, fixed, and then probed with anti‐rTsTryp immune serum, *T. spiralis*‐infected mouse serum or pre‐immune serum was used as a positive or negative control of primary antibodies and then stained using a CY3‐conjugated goat anti‐mouse IgG as a secondary antibody. Nuclei were counterstained blue with DAPI. Scale bar: 5 μm.

IIFA was performed to characterize the combination of rTsTryp with the cells. We observed that after rTsTryp incubation, bright red fluorescence expressed on the macrophage surface was detected by using anti‐rTsTryp serum and infection serum (Figure 1). The results indicated that rTsTryp was capable of binding to macrophages.

### 3.3. rTsTryp Promotes RAW264.7 M1 Polarization Through Activating NF‐κB Pathway

**Figure 2:**
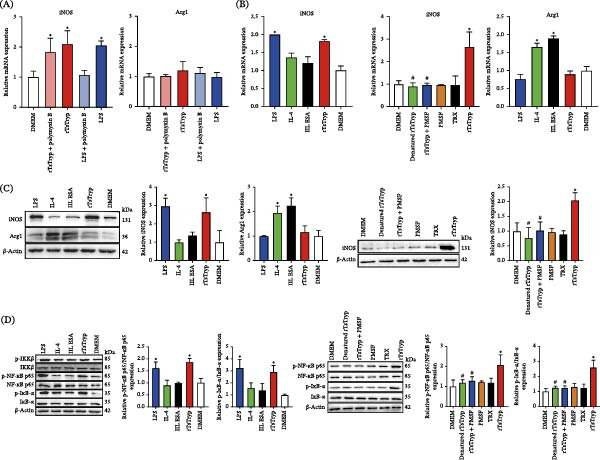
Expression level of iNOS, Arg‐1, and NF‐κB pathway proteins in rTsTryp‐treated RAW264.7 macrophages. A: qPCR assay of endotoxin removal efficiency of rTsTryp. B: qPCR analysis of mRNA levels of iNOS and Arg‐1. C: Western blotting of expression of both iNOS and Arg‐1. D: Western blotting of NF‐κB pathway protein levels. DMEM was served as a negative control and β‐actin as an internal control. Every assay had triplicates. Data were analyzed by one‐way ANOVA,  ^∗^ *p* < 0.05 in contrast to the DMEM group; data were analyzed by Student’s *t*‐test, ^#^ *p* < 0.05 relative to the rTsTryp group.

qPCR results of the polymyxin B neutralization experiment for endotoxin removal detection showed that iNOS expression level had no significant differences between the rTsTryp and rTsTryp + polymyxin B groups (*p* > 0.05), but iNOS expression in the two groups was markedly higher than in the DMEM group (*F* = 8.935, *p*  < 0.005). But iNOS expression level had an obvious differences between LPS and LPS +polymyxin group (*t*= 8.645, *p* < 0.005) (Figure 2A). The results indicated that the endotoxin in rTsTryp was successfully eliminated. qPCR analysis also showed that stimulation of RAW264.7 macrophages with rTsTryp significantly upregulated iNOS transcript levels, in relative to the DMEM control (*F*_iNOS_ = 43.19, *p*  < 0.001) (Figure 2B). No significant differences of iNOS transcription levels were observed among the TRX, denatured rTsTryp, rTsTryp + PMSF, PMSF, and DMEM groups (*p* > 0.05), but the iNOS transcription level of the rTsTryp + PMSF group was significantly declined relative to the rTsTryp group (*t* = 4.153, *p*  < 0.05). However, treatment with IL‐4 or IIL ESA markedly enhanced Arg‐1 transcript levels (*F*_Arg1_ = 72.52, *p*  < 0.001). These findings indicated that rTsTryp evidently promoted the macrophage M1 polarization, and the rTsTryp driving M1 polarization was dependent on its enzymatic activity.

Western blot analysis demonstrated that rTsTryp stimulation significantly upregulated iNOS expression in RAW264.7 cells relative to the DMEM group (*F*_iNOS_ = 10.41, *p* = 0.0014) (Figure 2C). The iNOS protein expression levels of the TRX, denatured rTsTryp, rTsTryp + PMSF, PMSF, and the DMEM group had no obvious difference (*p* > 0.05), and the iNOS protein level of the rTsTryp + PMSF group was clearly lower than that of the rTsTryp group (*t* = 4.153, *p*  < 0.05). In contrast, treatment with IIL ESA enhanced Arg‐1 expression level (*F*_Arg1_ = 17.41, *p* = 0.0002), but did not have any significant effect on iNOS protein expression. Moreover, Western blot analysis also revealed that stimulation of RAW264.7 cells with rTsTryp did not obviously alter the total protein expression levels of IKKβ, IκB‐α, or NF‐κB p65 (*p* > 0.05). However, rTsTryp significantly increased the phosphorylation levels of IκB‐α and NF‐κB p65 (*F*_p-NF-κB p65/NF-κB p65_ = 16.98, *p*  < 0.001; *F*_p-IκB-α/IκB-α_ = 11.08, *p*  < 0.001) (Figure 2D), the IKKβ phosphorylation level did not evidently change (*F* = 0.05535, *p* = 0.9934). The phosphorylation levels of IκB‐α and NF‐κB p65 in the TRX, denatured rTsTryp, rTsTryp + PMSF, PMSF, and DMEM groups had no obvious differences (*p* > 0.05). But the phosphorylation level of IκB‐α and NF‐κB p65 in the PMSF + rTsTryp group was prominently lower than in the rTsTryp group (*t*_p−NF−κB p65_ = 3.069, *t*_p−IκB−α_ = 4.641, *p*  < 0.05). Collectively, these results demonstrated that rTsTryp promotes macrophage M1 polarization via activation of the canonical NF‐κB signaling pathway, and this function of rTsTryp driving M1 polarization is related to its enzymatic activity.

### 3.4. rTsTryp Upregulates M1 Marker CD86 Expression in the Macrophages

**Figure 3:**
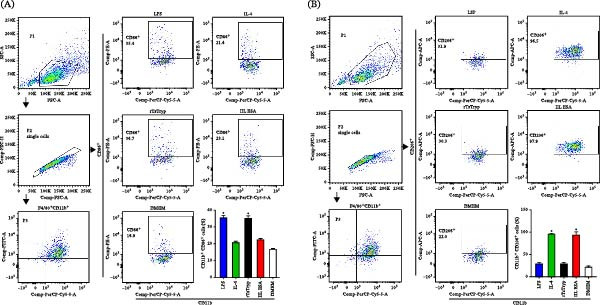
Flow cytometry of rTsTryp driving RAW264.7 M1 polarization. The effect of rTsTryp on CD86 (M1 marker) expression in RAW264.7 cells was assessed. LPS was served as an M1 positive control, and macrophages were gated as F4/80^+^CD11b^+^ populations. The effect of rTsTryp on CD206 (M2 marker) expression was also evaluated. IL‐4 was used as the M2 positive control, with F4/80^+^CD11b^+^ cells defined as macrophages. A: Black box exhibited the M1 cells (F4/80^+^, CD11b^+^, and CD86^+^), and percentage of CD86‐positive cells was presented in a bar graph. B: Black box showed M2 cells (F4/80^+^, CD11b^+^, and CD206^+^), and the percentage of CD206‐positive cells was presented in a bar graph. Each test had a triplicate. Data were analyzed with one‐way ANOVA,  ^∗^ *p* < 0.05 compared to the DMEM control group.

Flow cytometry results showed that RAW264.7 cells were gated as F4/80^+^ and CD11b^+^ populations, with CD86^+^ cells being identified as M1 macrophages and CD206^+^ cells as M2 macrophages. Treatment with rTsTryp significantly increased the proportion of CD86^+^ cells (*F* = 251.7, *p*  < 0.0001) (Figure 3A), while IIL ESA treatment markedly elevated the percentage of CD206^+^ cells (*F* = 344.6, *p*  < 0.0001) (Figure 3B). Combined with results from qPCR, Western blotting, and flow cytometry, these data demonstrated that rTsTryp drove RAW264.7 macrophage M1 polarization, whereas IIL ESA induced macrophage M2 polarization.

### 3.5. rTsTryp Upregulated M1‐Related Cytokine Expression

**Figure 4:**
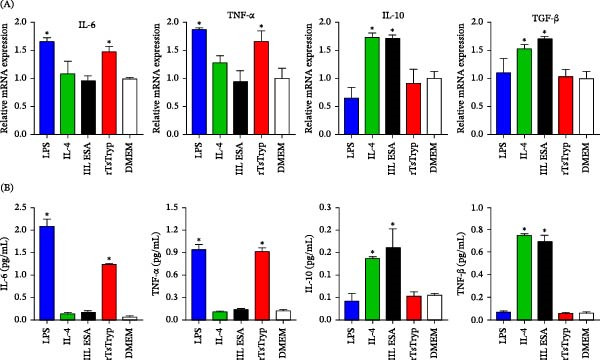
Expression levels of cytokines in RAW264.7 cells following rTsTryp stimulation. A: qPCR analysis of transcription levels of IL‐6, TNF‐α, IL‐10, and TGF‐β. Every assay had three replicates. B: ELISA analysis of expression levels of IL‐6, TNF‐α, IL‐10, and TGF‐β. All samples were assayed in duplicate. Data were analyzed with one‐way ANOVA,  ^∗^ *p* < 0.05 in contrast to the DMEM group.

qPCR results displayed that stimulation of RAW264.7 cells with rTsTryp obviously upregulated the transcript level of the proinflammatory cytokines (IL‐6 and TNF‐α), compared to the DMEM group (*F*_IL−6_ = 22.29, *F*_TNF-α_ = 21.87, *p*  < 0.001) (Figure 4A). These cells treated with IIL ESA exhibited markedly increased transcript level of anti‐inflammatory cytokines (IL‐10 and TGF‐β) (*F*_IL−10_ = 30.53, *F*_TGF-β_ = 16.74, *p*  < 0.001). But rTsTryp did not have any significant effect on the transcription of anti‐inflammatory cytokines (*p* > 0.05).

ELISA results demonstrated that the content of IL‐6 and TNF‐α was distinctly increased in the rTsTryp groups compared to the DMEM group (*F*_IL−6_ = 452.8, *F*_TNF-α_ = 434.1 *p*  < 0.0001) (Figure 4B). But the content of IL‐10 and TGF‐β in the rTsTryp groups showed no notable changes in comparison with the DMEM group (*p* > 0.05). But IIL ESA markedly increased expression levels of IL‐10 and TGF‐β relative to DMEM control (*F*_IL−10_ = 22.54, *F*_TGF-β_ = 601.0 *p*  < 0.0001). These findings indicated that rTsTryp promoted M1 polarization, while IIL ESA caused macrophage M2 polarization.

### 3.6. Inhibitor PDTC Suppressed rTsTryp‐Induced M1 Polarization and NF‐κB Pathway Activation in Macrophages

**Figure 5:**
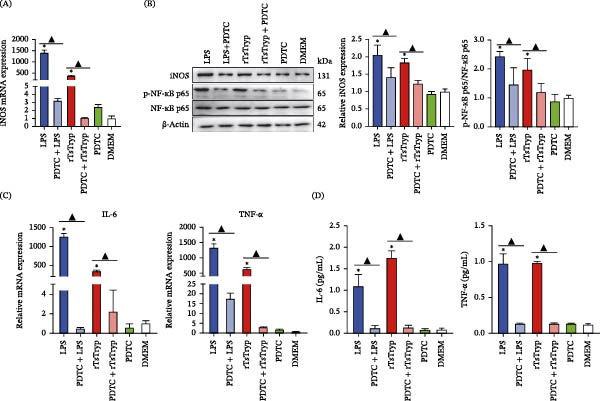
NF‐κB inhibitor PDTC suppressed rTsTryp‐induced RAW264.7 macrophage polarization and proinflammatory cytokine production. A: qPCR analysis of PDTC’s inhibitory effect on rTsTryp‐induced iNOS mRNA levels. B: Western blotting of protein expression of iNOS, p‐NF‐κB p65/NF‐κB p65. C: qPCR analysis of mRNA levels of proinflammatory cytokines (IL‐6 and TNF‐α) in PDTC‐pretreated and rTsTryp‐incubated RAW264.7 macrophages. D: ELISA results of proinflammatory cytokine (IL‐6 and TNF‐α) secretion in PDTC‐pretreated and rTsTryp‐incubated RAW264.7 macrophages. Data were analyzed with one‐way ANOVA, ^∗^ *p* < 0.05 compared with the DMEM group; data were analyzed with Student’s *t*‐test, ▲*p* < 0.05 between two groups.

The suppressive role of NF‐κB pathway inhibitor PDTC on rTsTryp‐induced RAW264.7 M1 polarization was evaluated using qPCR. Collected data revealed that PDTC pretreatment markedly abolished and declined the rTsTryp‐upregulated iNOS transcript level compared to the rTsTryp group (*t*_iNOS_ = 11.94, *p* = 0.0003) (Figure 5A). Western blot demonstrated that pretreatment of macrophages with PDTC notably diminished iNOS expression and decreased the p‐NF‐κB p65 level compared to the rTsTryp group (*t*_iNOS_ = 7.197, *p* = 0.002; *t*_p-NF-κB p65/NF-κB p65_ = 2.817, *p* = 0.048) (Figure 5B). These results demonstrated that NF‐κB pathway inhibitor PDTC suppressed M1 polarization and activation of the NF‐κB pathway in rTsTryp‐treated macrophages and further verified that rTsTryp‐induced M1 polarization of macrophages by activating the NF‐κB signaling pathway.

### 3.7. PDTC Inhibited Expression Levels of rTsTryp‐Induced Proinflammatory Cytokine

qPCR results showed that PDTC pretreatment significantly decreased transcript levels of IL‐6 and TNF‐α compared with the rTsTryp group (*t*_IL−6_ = 11.96, *p* = 0.0003; *t*_TNF-α_ = 20.79, *p*  < 0.0001) (Figure 5C). ELISA results demonstrated that PDTC pretreatment also markedly suppressed the production of IL‐6 and TNF‐α relative to the rTsTryp group (*t*_IL−6_ = 17.05, *p* = 0.0003; *t*_TNF-α_ = 70.48, *p*  < 0.0001) (Figure 5D). These findings further confirmed that the rTsTryp‐induced M1 polarization and upregulated generation of proinflammatory factors (IL‐6 and TNF‐α) through activating the NF‐κB pathway.

### 3.8. rTsTryp Increased NO Production

The Griess assay was applied to evaluate the NO content in rTsTryp‐stimulated RAW264.7 cells. A calibration curve for NO levels was generated according to absorbance readings at 540 nm of serial dilutions of NaNO_2_ (Supporting Information 2: Figure S2A). Results from the Griess assay showed that the NO concentration in cell culture supernatant exhibited markedly increased ZO secreations in the rTsTryp group compared with the DMEM control group (*F* = 1393, *p*  < 0.001) (Supporting Information 2: Figure S2B). Furthermore, when RAW264.7 cells were pretreated by PDTC, the rTsTryp‐induced NO production was markedly suppressed (*t* = 62.74, *p*  < 0.0001) (Supporting Information 2: Figure S2C). These findings suggested that rTsTryp‐enhanced macrophage NO secretion via activating the NF‐κB pathway.

### 3.9. PDTC Inhibiting NF‐κB Pathway Mitigated the rTsTryp‐Enhanced Macrophage Cytotoxicity

**Figure 6:**
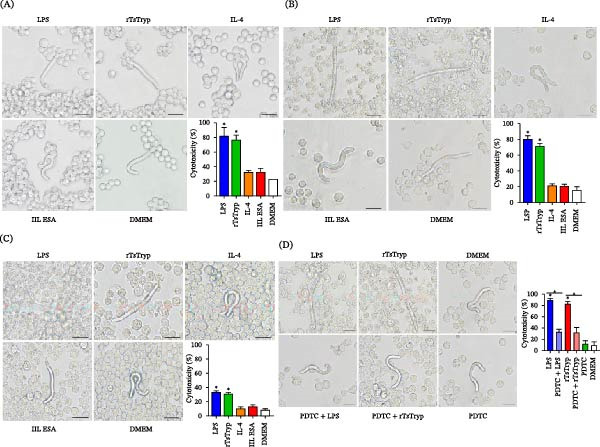
ADCC of rTsTryp‐induced M1‐polarized macrophages for killing *T. spiralis* newborn larvae. A‐C: RAW264.7 cells were first stimulated with rTsTryp for 24 h, followed by a 36 h co‐incubation with NBL and *T. spiralis*‐infected serum (A), rTsTryp‐immunized serum (B), or normal serum (C). D: PDTC inhibited macrophages’ cytotoxicity. Cytotoxicity was calculated as follows: Cytotoxicity (%) = (Number of dead NBL + macrophage‐adhered NBL)/total NBL number × 100%. Data were analyzed with one‐way ANOVA,  ^∗^ *p* < 0.05 compared to DMEM group; Data were analyzed by Student’s *t*‐test, ▲*p* < 0.05 between two groups. The data were also analyzed with the chi‐square test. Scale bar: 50 μm.

The ADCC results demonstrated that when three kinds of serum were used, stimulation with rTsTryp significantly enhanced the cytotoxic effect of RAW264.7 cells killing NBL compared to the DMEM control group (*χ* ^2^_infection serum_ = 406.6, *χ* ^2^_immune serum_ = 478.3, *χ* ^2^_normal serum_ = 133.0, *p*  < 0.0001) (Figure 6A‐6C). When infection serum was used, the cytotoxic effect of RAW264.7 cells killing NBL was substantially decreased relative to the rTsTryp alone group following pretreatment with PDTC (*χ^2^* = 236.72, *p*  < 0.0001) (Figure 6D), indicating that the rTsTryp‐enhanced macrophage ADCC is mediated via triggering the canonical NF‐κB pathway.

### 3.10. rTsTryp Promoted NF‐κB p65 Nuclear Translocation

IIFA findings indicated that following incubation with rTsTryp, NF‐κB p65 in RAW264.7 cells was gradually translocated into the cellular nucleus. But no obvious NF‐κB p65 nuclear translocation was detected in TRX, denatured rTsTryp, rTsTryp + PMSF, PMSF, and DMEM groups (Supporting Information 3: Figure S3). The findings further verified that the rTsTryp driving macrophage M1 polarization and enhancing ADCC are related to the NF‐κB pathway.

## 4. Discussion

After *T. spiralis* infection, the host rapidly initiates an immune defense dominated by inflammatory responses. Although this response is beneficial to restrict parasite dissemination, excessive or prolonged immune activation is often accompanied by substantial cellular and tissue damage. To ensure long‐term survival and successful completion of its life cycle within the host, *T. spiralis* actively modulates host immune responses by secreting multiple immunoregulatory molecules, thereby shaping a relatively stable immune microenvironment favorable for parasite settlement and persistence [41, 42].

Previous studies demonstrated that *T. spiralis* ESA exerts a pivotal role in regulating innate immune response. *T. spiralis* ESA at different developmental stages exhibited distinct immunomodulatory properties. For instance, under LPS stimulation, the ML ESA markedly suppressed the secretion of macrophage proinflammatory cytokines (IL‐1, IL‐6, and IL‐12) and impeded activation of ERK1/2 and p38 MAPK signaling pathways. The only adult worm ESA treatment enhanced the production of immunosuppressive or tissue repair‐associated molecules (IL‐10, TGF‐β, and Arg‐1) in macrophages [43, 44], suggesting their potential to induce immune tolerance and anti‐inflammatory responses. Adult‐derived ESA promoted macrophage polarization toward the M2 phenotype, as demonstrated by a marked upregulation of the abundance of CD206 and Arg‐1, and effectively mitigated dextran sulfate sodium (DSS)‐evoked colitis in mice [45]. Moreover, TsTryp used in this study has been identified as a highly expressed ESA at the IIL and adult stages of *T. spiralis* infection, implying that it may serve as a key regulator in early interactions of parasite and host [11, 13, 46].

Macrophages play a key role in identifying and eliminating pathogens and modulating inflammatory responses. Macrophages undergo polarization into two evident different phenotypes upon diverse stimuli: the classically activated M1 macrophage and the alternatively activated M2 macrophage. M1 macrophage plays direct cytotoxicity on pathogens and strengthened antiparasitic immunity, whereas M2 macrophage is involved in tissue repair and immune modulation and the immune escape of the pathogens [45, 47]. In the immune response against *T. spiralis* infection, macrophage polarization is highly related to the worm clearance: M1‐polarized macrophages increased the ADCC destroying NBL, while M2 polarized macrophages promoted the worm colonization in the host [16].

In this investigation, we aimed to explore the rTsTryp regulatory function on macrophage polarization; endotoxin‐free rTsTryp was utilized to stimulate RAW264.7 macrophages in vitro. LPS and IL‐4 were, respectively, served as a positive control of M1/M2 macrophage polarization, while *T. spiralis* IIL ESA served as a parasite‐derived protein control [48]. Immunofluorescence staining showed that rTsTryp could specifically bind to macrophages, indicating a direct interaction between rTsTryp and the host macrophages [49]. To further assess the impact of rTsTryp on macrophage polarization, the expression level of M1/M2 effector molecules was also ascertained. The results displayed that rTsTryp markedly induced the level of the macrophage M1‐specific molecule iNOS, but it had no apparent effect on the content of the M2 marker Arg‐1, indicating that rTsTryp drives macrophage polarization toward the M1 phenotype. Additionally, rTsTryp treatment markedly increased the transcription levels of multiple M1‐associated genes (IL‐6, TNF‐α, CD86, and iNOS) and the expression level of surface marker CD86 in RAW264.7 macrophages. rTsTryp also markedly increased the production of NO and enhanced macrophage’s cytotoxicity, killing NBL via activation of the NF‐κB signaling pathway [50].

Furthermore, rTsTryp also significantly increased the abundance of p‐NF‐κB p65 and p‐IκB‐α in macrophages. However, pretreatment of macrophages with the NF‐κB inhibitor PDTC significantly reduced rTsTryp‐increased iNOS expression. The abundance of p‐NF‐κB p65 and p‐IκB‐α, proinflammatory cytokines (IL‐6 and TNF‐α) in PDTC‐pretreated macrophages evidently declined, suggesting that PDTC inhibiting the NF‐κB pathway effectively counteracted this M1 polarization [51–53]. Additionally, the abundance of iNOS, p‐IκB‐α, and p‐NF‐κB p65 in the rTsTryp+PMSF group was visibly declined relative to the rTsTryp group, and rTsTryp facilitated NF‐κB p65 nuclear translocation of rTsTryp‐treated macrophages. These findings indicated that rTsTryp directly drove macrophage M1 polarization in vitro by activating the NF‐κB pathway, and this function of rTsTryp driving M1 polarization is linked to its enzymatic activity. These observations were accordant with the macrophage M1 polarization observed at the early stage of *T. spiralis* infection and M1 polarization induced by recombinant *T. spiralis* cathepsin L (rTsCatL) and recombinant galactoside‐binding lectin family protein (rTsGLFP) [25, 40, 54]. During *T. spiralis* infection, M1 macrophage–derived proinflammatory cytokines (e.g., TNF‐α and IL‐6) occupy a dominant position in initiating and amplifying inflammatory responses by promoting immune cell activation and recruitment, thereby facilitating effective clearance of invading pathogens [55, 56].

To further investigate the capacity of rTsTryp‐stimulated macrophages killing the larvae, anti‐*T. spiralis* antibody‐mediated ADCC to kill the NBL was conducted; microscopic observation revealed that rTsTryp stimulation significantly enhanced the RAW264.7 macrophages’ cytotoxicity, killing NBL. However, PDTC pretreatment markedly attenuated the rTsTryp‐enhanced ADCC. These observations are consistent with our earlier results of rTsTryp activating the NF‐κB pathway, upregulating the level of M1 markers (iNOS and CD86), and driving M1 phenotypic polarization of macrophages. The increase of proinflammatory cytokines produced from M1 macrophages promotes a gut inflammatory reaction that accelerates intestinal expulsion of worms from the gut, decreases the production of the NBL, and thereby reduces ML burdens and alleviates the infection [17]. Immune response triggered at the gut stage of *T. spiralis* infection was directed to the NBL, and the macrophage cytotoxicity killing NBL was evidently strengthened [57]. Previous investigations have demonstrated that the purinergic P2X7 receptor (P2X7R) and NLRP3 inflammasome are closely associated with intestinal immune responses to *Trichinella* infection. *Trichinella* infection upregulates the P2X7R expression and triggers NLRP3 inflammasome activation in murine macrophages. Blocking the P2X7R receptor suppressed the activation of NLRP3/IL‐1b via NF‐κB and decreased the macrophage capacity of killing NBL [15]. Moreover, the mice treated with the NLRP3 inhibitor MCC950 showed that NLRP3 enhanced the Th1 immunity in adult and NBL stages and weakened the Th2 immunity in the ML stage. NLRP3 promoted the production of proinflammatory cytokine IFN‐γ and inhibited the release of anti‐inflammatory cytokine IL‐4. The NLRP3 inhibitor also significantly reduced the adult and ML burden at 7 and 35 dpi in inhibitor‐treated infected mice [58]. These findings suggested that the P2X7R receptor and NLRP3 inflammasome have an important role for protective immunity at the enteral stage of the *Trichinella* infection. Additionally, the macrophages could also destroy the NBL directly by producing and liberating NO [59].

Furthermore, when normal murine serum was used, rTsTryp‐treated RAW264.7 macrophages also had the capacity to kill NBL, suggesting that rTsTryp‐stimulated macrophages exerted cytotoxic effects against NBL in the absence or presence of specific antibodies. However, the larval death percentage was lower than those obtained with infection serum and immune serum. This phenomenon is consistent with the biological characteristics of normal serum with basal innate immune activation, while infection and immune serum are enriched with specific antibodies and other immune‐activating factors [60]. Previous studies showed that intestinal lamina propria cells (mainly neutrophils, eosinophils, and macrophages) from *T. spiralis*‐infected rats at 3 dpi were capable of killing the NBL in the presence of sera from noninfected rats, implying that the intestine serves as a primary site of migrating larva destruction and testifying that not all NBLs liberated from the intestine can successfully migrate to the skeletal muscles [57].

However, this study still has some limitations. The specific receptor mediating TsTryp binding on the macrophage surface remains unidentified, and the kinds and properties of the receptors require to be identified by the co‐immunoprecipitation assays, pull‐down assays, and mass spectrometry analysis in further studies [61]. Additionally, whether TsTryp drives the in vivo macrophage polarization and its role mechanism remain unclear. These issues need to be investigated in future studies.

## 5. Conclusions

rTsTryp specifically bound to macrophages and activated the NF‐κB signaling pathway, as manifested by the obviously increased phosphorylation level of NF‐κB p65 and IκB‐α. The NF‐κB pathway activation markedly elevated the levels of M1 markers (iNOS and CD86) and increased the content of proinflammatory cytokines (IL‐6 and TNF‐α) and NO. rTsTryp also significantly enhanced the RAW264.7 macrophage’s cytotoxicity, killing NBL. Pretreating macrophages with the NF‐κB‐specific inhibitor PDTC markedly suppressed the macrophage M1 polarization and the capacity to kill NBL. Collectively, these findings provided new insights into the immune modulatory function of TsTryp in *T. spiralis* infection.

## Author Contributions

Jing Cui and Zhong Quan Wang designed and supervised the research. Pei Kun Cong, Yao Zhang, Ru Zhang, Xin Zhuo Zhang, Ruo Dan Liu, Shao Rong Long, Jia Xu, Jing Cui, and Zhong Quan Wang carried out the experiments. Pei Kun Cong analyzed the data. Pei Kun Cong, Jing Cui, and Zhong Quan Wang drafted the initial manuscript. Pei Kun Cong and Ruo Dan Liu performed the analysis of macrophage M1/M2 polarization.

## Funding

This research was funded by grants from the National Natural Science Foundation of China (Grants 82372276 and 82302565).

## Disclosure

All authors have read, reviewed, and approved the final version of this manuscript.

## Ethics Statement

All experimental schemes were authorized by the Life Science Ethics Committee of Zhengzhou University (Number ZZUIRB GZR 2023‐1397).

## Conflicts of Interest

The authors declare no conflicts of interest.

## Supporting Information

Additional supporting information can be found online in the Supporting Information section.

## Supporting information


**Supporting Information 1** Figure S1: CCK‐8 assay was carried out to evaluate the effects of rTsTryp and IIL ESA on RAW264.7 cell viability. Cells were treated with various doses of rTsTryp or IIL ESA for 24 and 48 h.  ^∗^ *p* < 0.05 compared with the blank control group (0 µg/mL).

**Supporting Information 2** Figure S2: Griess assay of rTsTryp‐induced NO production in RAW264.7 culture supernatant. A: representing the standard calibration curve for nitric oxide (NO) concentration measurement. B: displaying NO secretion levels from macrophage populations cultured with recombinant TsTryp (rTsTryp) protein over a 24‐h incubation period. C: indicating NO release from PDTC‐pretreated macrophages following rTsTryp stimulation for 24 h. Lipopolysaccharide was served as the positive control group, while interleukin‐4 (IL‐4) was set as the negative control group.  ^∗^ *p* < 0.001 vs. DMEM control group; #*p* < 0.01 for pairwise intergroup comparisons.

**Supporting Information 3** Figure S3: IIFA results of rTsTryp promoting NF‐κB p65 nuclear translocation. RAW264.7 cells were fixed after rTsTryp stimulation and stained by anti‐NF‐κB p65 antibody. Subsequently, CY3‐goat anti‐mouse IgG conjugate was used for the secondary antibody, and cell nuclei were dyed blue by DAPI. Scale bar: 20 μm.

## Data Availability

Raw data generated in this study are available from the corresponding author upon reasonable request.
