# Supplementary figures and images for: A Trichinella spiralis Trypsin Drives Macrophage M1 Polarization and Strengthens Cytotoxicity Killing Larvae via Activating the NF‐κB Pathway
Source: Transbound Emerg Dis. 2026 Jul 3;2026:3937439. doi: 10.1155/tbed/3937439 (PMC13329453; doi:10.1155/tbed/3937439)

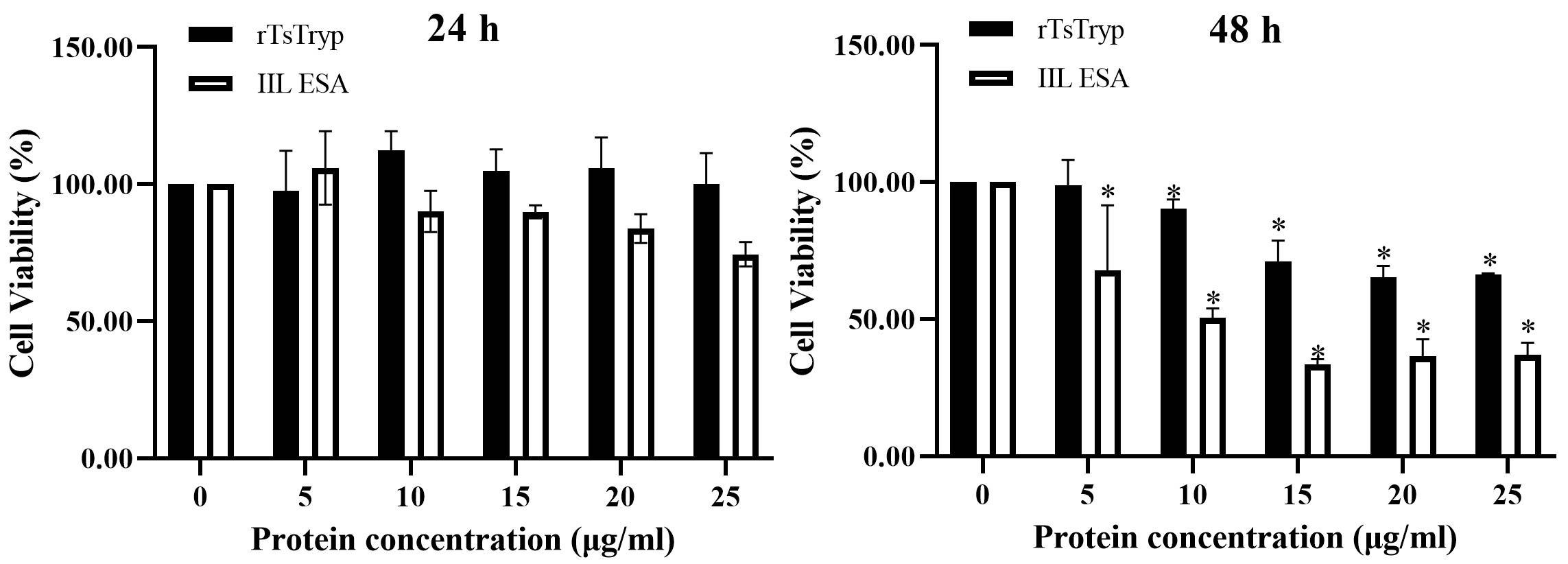

Supplement: Supplementary file 1 — Supporting Information 1 Figure S1: CCK‐8 assay was carried out to evaluate the effects of rTsTryp and IIL ESA on RAW264.7 cell viability. Cells were treated with various doses of rTsTryp or IIL ESA for 24 and 48 h. ∗ p < 0.05 compared with the blank control group (0 µg/mL). [file TBED-2026-3937439-s002.jpg]

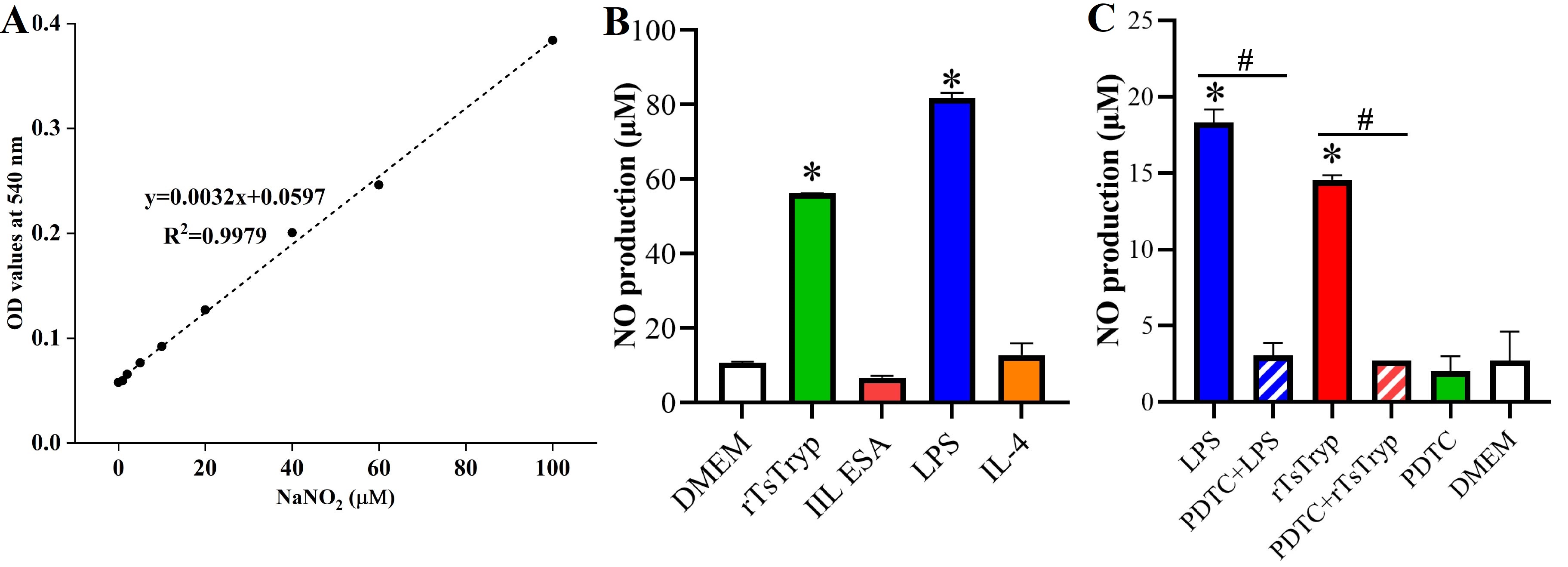

Supplement: Supplementary file 2 — Supporting Information 2 Figure S2: Griess assay of rTsTryp‐induced NO production in RAW264.7 culture supernatant. A: representing the standard calibration curve for nitric oxide (NO) concentration measurement. B: displaying NO secretion levels from macrophage populations cultured with recombinant TsTryp (rTsTryp) protein over a 24‐h incubation period. C: indicating NO release from PDTC‐pretreated macrophages following rTsTryp stimulation for 24 h. Lipopolysaccharide was served as the positive control group, while interleukin‐4 (IL‐4) was set as the negative control group. ∗ p < 0.001 vs. DMEM control group; #p < 0.01 for pairwise intergroup comparisons. [file TBED-2026-3937439-s003.jpg]
